# Study of Crocin & Radiotherapy-induced Cytotoxicity and Apoptosis in the Head and Neck Cancer (HN-5) Cell Line

**Published:** 2017

**Authors:** Vahid Vazifedan, Seyed Hadi Mousavi, Javad Sargolzaei, Shokouhozaman Soleymanifard, Azar Fani Pakdel

**Affiliations:** a *Medical Toxicology Research Center, Mashhad University of Medical Sciences, Mashhad, Iran.*; b *Department of Biology, Mashhad Branch, Islamic Azad University, Mashhad, Iran . *; c *Department of Biochemistry, Institute of Biochemistry and Biophysics, University of Tehran, Tehran, Iran. *; d *Omid Hospital Medical Physics Department, Mashhad University of Medical Sciences, Mashhad, Iran.*; e *Department of Medical Physics, School of Medicine, Mashhad University of Medical Sciences, Mashhad, Iran.*; f *Cancer Research Center, Mashhad University of Medical Sciences, Mashhad , Iran.*

**Keywords:** HN-5, Crocin, Cytotoxicity, Apoptosis, Radiotherapy

## Abstract

Malignant tumors of head and neck carcinomas are the sixth most common type of cancer. Current systemic therapies for cancer show side effects in normal tissues and short-term efficacy due to drug resistance. Consequently, there is much interest in identifying new drugs for cancer treatment. Crocin (an active ingredient of saffron) has been shown to have cytotoxic effects on cancer cell lines. Chemo radiotherapy is the standard treatment for head and neck cancer. In the present study, the cytotoxic effects, inducing apoptosis and the radiation sensitivity of crocin were evaluated in the head and neck cancer cell line (HN-5). HN-5 cells were cultured in a DMEM medium and incubated with different concentrations of crocin (12.5-1000 µg/mL). They were exposed to 2 Gy γ-rays. Cell viability was quantified by the MTT assay. Apoptotic cells were determined using PI staining of DNA fragmentation by flowcytometry (sub-G1 peak). Crocin decreased cell viability in HN-5 cells in a time and concentration dependent manner. Crocin also induced a sub-G1 peak in the flowcytometery histogram of treated cells compared with the control, suggesting that apoptotic cell death is caused by its toxicity. Crocin was also shown to sensitize cells to radiation-induced toxicity and apoptosis. The simultaneous use of crocin and radiation therefore increases radiation sensitivity and cell death. Thus, after further study crocin can be considered as a potential drug and sensitizer in cancer treatment.

## Introduction

Head and neck cancer is the sixth most common cancer in the world (1). About 3.2 percent of all cancers in the United States originate from head and neck. In 2012, out of more than the 40,000 new cases of head and neck cancers were diagnosed and 7,850 related deaths were expected (2). Head and neck squamous cell carcinomas (HNSCC), of the pharynx and oral cavity make an estimated 2.5 percent of cancer diagnosis in the United States (3). Two important risk factors for developing HNSCC include tobacco and alcohol (4). Actually, a recent survey showed that, of 16,000 controls and more than 11,000 cases, 72 percent of head and neck cancer cases could be ascribed to the use of alcohol or tobacco (5). The most common accepted standard therapy for advanced stages of HNSCC is combined radiotherapy and surgery (6, 7). *Crocus sativus *L. commonly known as saffron is an herb of the Iridaceae family. Its basic components are safranal, crocin, picrocrocin and crocetin (8). Crocin produces saffron’s color. This carotenoid inhibits the morphine withdrawal syndrome (9). In addition, pharmacological studies have shown that crocin can be used as a therapeutic agent. Its various effects, among others, are anti-nociceptive, anti-inflammatory (10, 11), anti-convulsant (12), anti-depressant (13), as an antidote activities (14-16), anti-atherogenic*, *anti-platelet, anti-oxidant (17)*, *neuron-protective*,* genoprotective (18), memory-improving (19, 20), aphrodisiac*, *hypolipidemic, anxiolytic-like (17) and radical scavenging (21).

It also has an eligibility significant anti-proliferation effect on pancreatic and bladder cancer (22), ovarian carcinoma cells, osteosarcoma, fibrosarcoma, leukaemia (23) and human colorectal cancer cells. Notably, crocin inhibits the growth of cancer cells. and but yet has no effect on normal cells (24). Crocin can induce gene expression in the T24 (transitional cell carcinoma of bladder) cell.

The effect of crocin and its radiosensetivity on head and neck cancer has not yet been investigated. In the present study, the cytotoxic and apoptogenic effects of crocin, combined with radiation, on head and neck squamous cell carcinoma are examined.

## Experimental

*Reagents*

The standard crocin was donated by Dr. Ahmad Mohajeri. Propidium iodide (PI), sodium citrate, 3-(4, 5-dimethylthiazol-2-yl)-2, 5-diphenyl tetrazolium (MTT) and Triton X-100 were purchased from Sigma. The DMEM and fetal bovine serum (FBS) were purchased from Gibco.

*Cell Culture Conditions*

HN-5 cells were cultured in a DMEM containing 10% FBS and 1% streptomycin and penicillin antibiotic. HN-5 cells were grown as monolayers in a 25-cm^2^ flask and were maintained in a humidified 5% CO_2_ and 95% air atmosphere at 37 °C.

*Determination of Cytotoxicity and Apoptosis by Using Crocin in Combination with Radiation*

Radiation therapy uses high-energy radiation to kill cancer cells and tumors (25). Gamma rays, x-rays, and charged particles are the types of radiation employed for cancer therapy. For radiotherapy in the current study, HN-5 cells were irradiated with γ-rays during exponential cell growth as monolayers in 96 and 24-well plates using a ^60^Co unit at a dose of 2 Gy (26). Cells were maintained in a DMEM with 10% FBS and 1% streptomycin and penicillin antibiotic during all radiation exposures. Cell viability was assessed 66 h (27) after radiation by the MTT test.

*MTT Assay*

For the MTT assay, the cell viability of cultured cells was determined by assaying the reduction of 3-(4, 5-dimethylthiazol-2-yl)-2, 5-diphenyl tetrazolium (MTT) assay. Briefly, HN-5 cells were cultured at an initial density of 5000 cells/well onto flat-bottomed 96-well culture plates and were allowed to grow 24 h. This was followed by a treatment with crocin (12.5–1000 μg/mL). After a set time (6 h, 48 h and 72 h), one of the two plates received 2 Gy γ-rays. Then, both plates (irradiated and non-irradiated) were incubated for 66 h. After removing the medium, the cells were then treated with a MTT solution (5 mg/mL in PBS) for 4 h. The resulting formazan was solubilized with DMSO (100 μL). Absorbance was measured at 570 nm (620 nm as a reference) by an ELISA reader (28).

*Propidium Iodide Staining*

The apoptotic cells were determined by the propidium iodide (PI) method of treating cells, followed by flowcytometery to find the so-called sub-G1 peak (29, 30). Briefly, HN-5 cells were adhered overnight in a 24-well plate at an initial density of 100,000 cells/well and then treated with crocin (12.5-500 μg/mL) for 24 h. They were then exposed to gamma rays (2 Gy). After 66 h, the cells were washed with PBS and resuspended in 750 μL of a hypotonic buffer (50 μg/mL PI in 0.1% sodium citrate and 0.1% Triton X-100) (27). HN5 cells were then incubated at 37 °C for 30 min before a flowcytometric analysis.

*Statistical Analysis*

Data were expressed as mean ± SEM. The test was performed using a one-way ANOVA, followed by the Bonferroni test for several comparisons. A probability level of P < 0.05 was considered statistically significant.

## Results and Discussion

Effect of Simultaneous Radiotherapy and Crocin on Cell Viability

**Figure 1:**
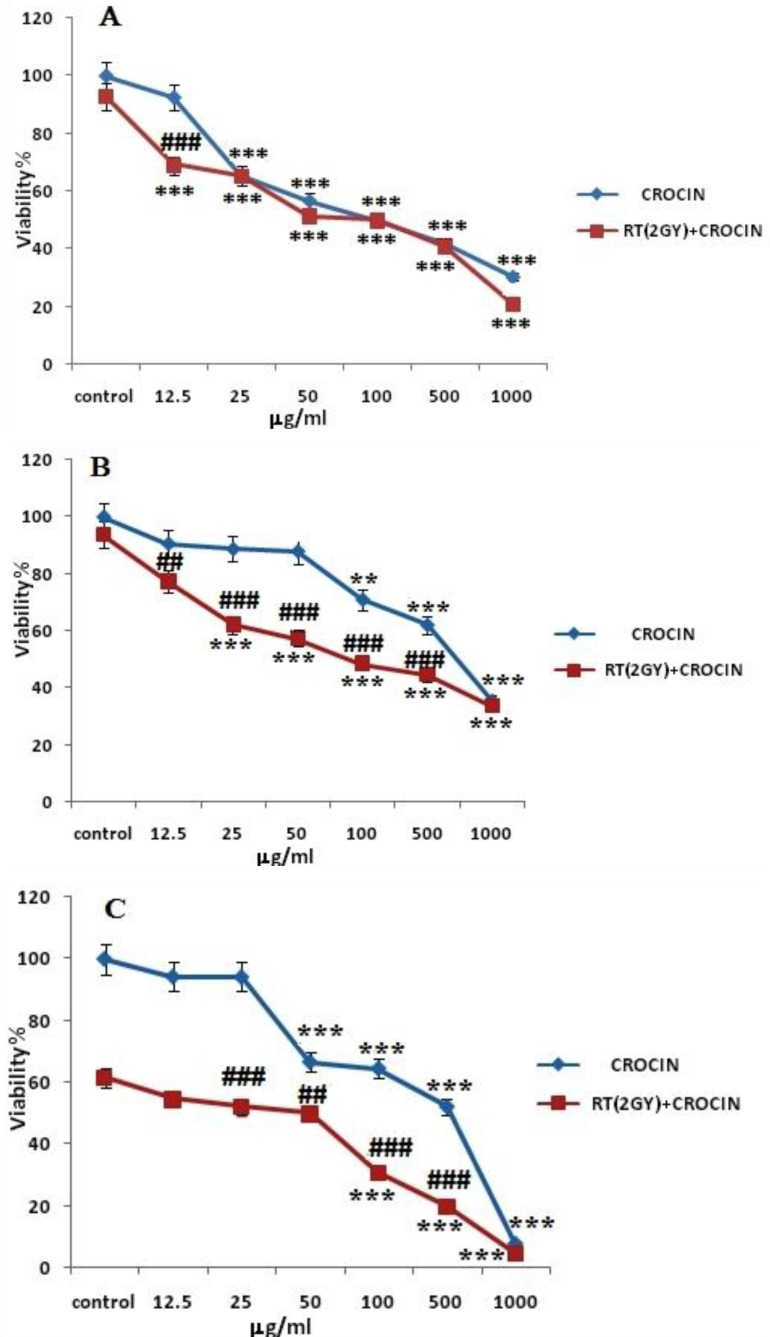
The effect radiation sensitivity and toxicity of crocin on HN-5 cell-line. Cells were treated with different concentrations of crocin for 6 h (A), 48 h (B), and 72 h (C) and were exposed to 2 Gy γ-rays. Cell viability was quantitated by the MTT assay. Results are mean ± SEM (*n *= 3). The asterisks are indicators of statistical differences when compared with the controls shown in the figure as * *P *< 0.05, ** *P *< 0.01, and *** *P *< 0.001. There were obtained separately at different time points.

HN-5 cells were incubated with different concentrations of crocin (12.5–1000 μg/mL) for 6, 48 and 72 h. Afterwards, they received 2 Gy γ-rays and were then incubated for 66 h. The results showed that crocin could decrease cell viability in HN-5 malignant cells in a time and concentration dependent manner (Figure 1). It was also found that Crocin could sensitize cells to radiation-induced toxicity (Table 2). In other words, crocin increased radiation sensitivity and cell death. 

*Role of Apoptosis*

**Figure 2:**
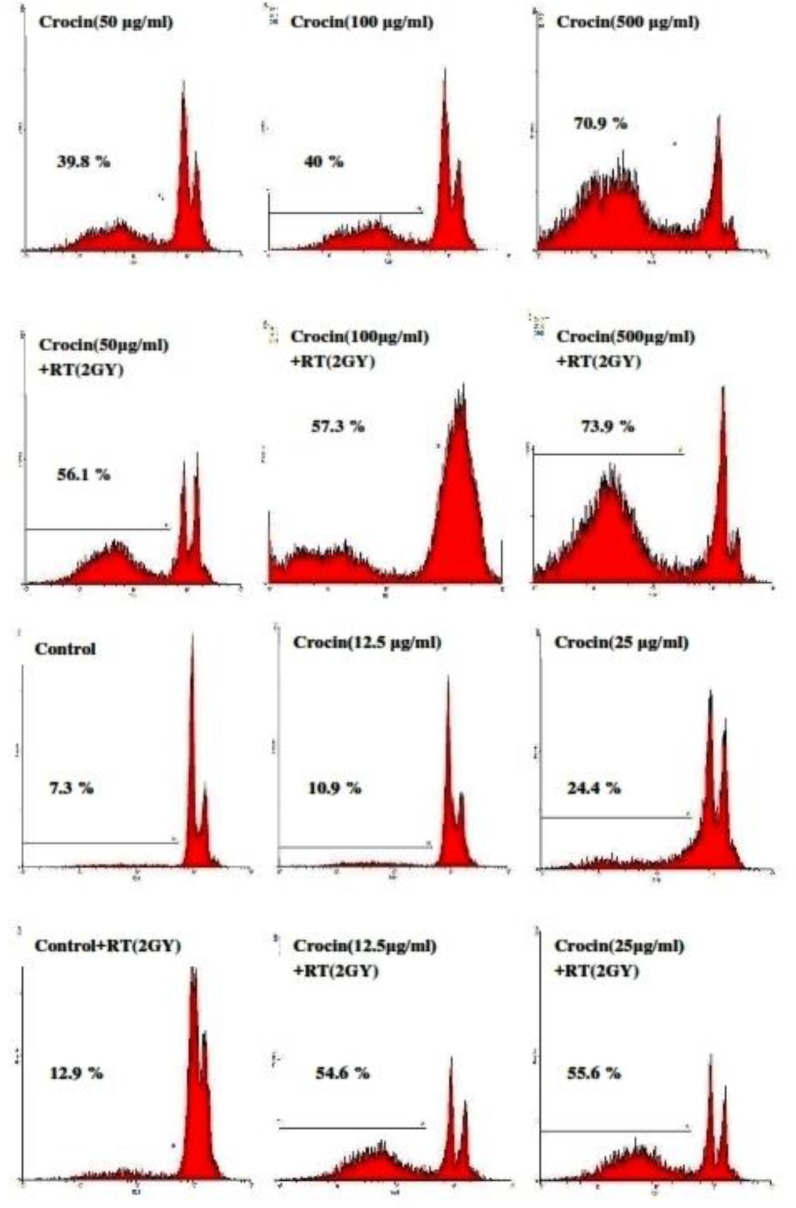
Flow cytometery histograms of apoptosis assays by the propidium iodide (PI) method in HN-5 cells after 66 h. HN-5 cells, which were treated with (12.5-500 µg/mL) of crocin and 2 Gy radiation, were affected in comparison with those that had not received radiation. A sub-G1 peak, as an indicator of apoptotic cells, was induced in crocin-treated HN-5 cells

The proportion of apoptotic cells was measured with a PI staining of DNA fragmentation by flowcytometry. Crocin (12.5-500 μg/mL) induced a sub-G1 peak in the flowcytometery histogram of treated cells when compared with the control, thus suggesting that apoptotic cell death is involved in its toxicity. Results indicate a crocin-induced apoptosis in a concentration-dependent manner. Crocin was also found to sensitize cells to radiation-induced apoptosis (Figure 2). Therefore, a simultaneous use of crocin and radiotherapy increases radiation sensitivity and cell death. The Percentage of apoptotic cells in HN-5 cells is presented in Table 1.

Cancer is a major health issue worldwide. Natural compounds have long been used to prevent and treat cancer. Thus, it is appropriate to develop these compounds as anti-cancer drugs (31). Anti-tumor drugs are known to regulate cell cycle progression, inhibit cell proliferation, and induce apoptosis in cancer cells (32). The two main ways to prevent tumor growth and its progression include the induction of cell death and the inhibition of cell growth (33, 34). Previous research suggests that saffron and its carotenoids display antitumor properties (35, 36). Several mechanisms to employ the anti-cancer properties of saffron and its components have been suggested, including the inhibition of nucleic acid, a free radical chain reaction, the effect of carotenoids on the expression of topoisomerase П, and the induction of apoptosis (37). Previously, Mousavi *et al*. demonstrated that saffron and its components exert an anti-cancer effect on HepG2 and HeLa cell lines (38). The cytotoxic effects of crocin have also been detected in different cell lines, including hepatocellular carcinoma (KIM-1), the acute promyelocytic leukemia cell line (HL-60), lymphoid leukemia cells, HeLa cells, and K562 (24, 39).

Different mechanisms for crocin-induced cytotoxicity have been proposed, such as G1-phase cell cycle arrest (24), regulation of the cell cycle which controls gene expression, and regulation of cyclin D1, Bcl-2, survivin and Bax expression (17).

To study the effect of this carotenoid on the induction of cell death and the inhibition of cell growth, the current study evaluated the effects of crocin on head and neck cancer cells. These cells were exposed to an increasing concentration of crocin for 6, 48, and 72 h, with cell viability quantitied by the MTT assay.

## P <0.05, ### P < 0.01 are compared to the counterpart concentration.

**Table 1:** The proportion of apoptosis in crocin-induced cytotoxicity in HN-5 cells. The cells were treated with different concentrations of crocin for 24 h. After 24 h, the cells were exposed to 2 Gy γ-rays and incubated for 66 h

	**Crocin concentration(μg/mL ) **	**Control **	**12.5**	**25**	**50**	**100**	**500**
**Apoptosis (%)**	**Crocin**	7.3	10.9	24.4	39.8	40	70.9
	**Crocin+RT (2GY)**	12.9	54.6	55.6	56.1	57.3	73.9

**Table 2:** Doses (µg/mL) inducing 50% cell growth inhibition (IC50) of crocin against HN-5 cell line. Cells were treated with different concentrations of crocin for 6, 48, and 72h and were exposed to 2 Gy γ-rays. Viability was quantitated by MTT assay

**IC50**	**6h (without RT) **	**6h (with RT)**	**48h (without RT)**	**48h (with RT)**	**72h (without RT)**	**72h (with RT)**
HN-5	534	131	580	145	483	97.8

The results confirmed that saffron-derived crocin conducts anti-proliferation and anti-carcinogenic activities. Based on Figures 1 and 2. cell viability in the HN-5 cell line decreased after exposure to crocin in a time and concentration dependent manner. Also, crocin was seen to induce apoptosis in a concentration-dependent manner.

The concomitant use of chemotherapeutic agents and radiotherapy on radiosensitivity in head and neck cancers have been studied (27). Mylin A. Torres *et al*. explored the effects of AC480 cells exposed to increasing concentrations of crocin for 6, 48, and 72 h, with cell viability quantified by an MTT assay.

The current study’s results confirmed that saffron-derived crocin has anti-proliferation and anti-carcinogenic properties. Based on Figures 1 and 2. cell viability in the HN-5 cell line decreased after exposure to crocin in a time and concentration dependent manner. Also, crocin: caused apoptosis in a concentration-dependent manner.

The concomitant use of chemotherapeutic agents and radiotherapy on radiosensitivity in head and neck cancers have been previously studied (27). Mylin A. Torres *et al*. analyzed the effects of AC480 combined with radiation of head and neck cancer. They found that the drug significantly enhanced the *in-vitro* radio sensitivity of the HN-5 cell line (27). Luka Milas *et al.* examined the use of a combined drug (C225 antibody) with radiation of head and neck cancer. They reported that the drug enhanced the response of these cells to radiation *in-vitro*; the enhancement was attributed to increased radiation-induced apoptosis (40).

In another work, Daisuke Sano *et al*. showed that vandetanib restores the sensitivity of head and neck cancer cells to cisplatin and radiation. They reported that vandetanib and cisplatin effectively radiosensitized head and neck squamous cell carcinoma both *in-vitro* and *in-vivo* (41). Since no information had been available on the effects of crocin combined with radiotherapy, the current study determined the effects of crocin, combined with radiation, on HN-5 cell line. It was confirmed that the concomitant use of this drug (crocin) and radiation on HN-5 cell line increases radiation sensitivity and radioresponse and cell death (Table 2). Crocin was also reported to have cytotoxic and anti-proliferation properties which work against HN-5 cell line.

In the present investigation, crocin-induced apoptosis was involved in the induction of cell death. Apoptosis is a gene-regulated phenomenon. It is induced by many chemotherapeutic agents used in cancer therapy (42, 43). Apoptosis is characterized by different morphological features, including cell and nuclear shrinkage blebbing, chromatin condensation, membrane and oligonucleosomal DNA fragmentation (44). According to previous reports, DNA fragmentation creats small fragments of DNA that can be eluted following incubation in a PI buffer. When stained with a quantitative DNA-binding dye, such as PI, the cells that have lost DNA will absorb less stain and will be visible on the left of the G1 peak. In the current study’s results, apoptosis was identifiable by the detection of a sub-G1 peak. Compared to the control crocin induced a sub-G1 peak in the flowcytometery histogram of treated cells, thus suggesting that apoptotic cell death is involved in its toxicity.

In addition, the present study reported that the sub-G1 peak in crocin-treated HN-5 cultures exposed to 2 Gy **γ**-rays was higher than those not exposed to **γ**-rays. It is notable that, the induction of apoptosis in tumor cells can be highly effective in the treatment of cancer and the prevention of its recurrence (29).

In summary, the present study is the first to show the cytotoxic effects of crocin on head and neck cancer. Crocin also sensitizes cells to radiation-induced toxicity, in which apoptosis (programmed cell death) plays an important role. Thus, after further pre-clinical and clinical studies, crocin can be considered as a potential drug and radiosensitizer in head and neck cancer treatment.

Studies demonstrate that crocin inhibits the proliferation of HN-5 cells. Crocin can also sensitize cells to radiation-induced toxicity and apoptosis. These results suggest that crocin can potentially be developed as a new drug with a high efficacy, low toxicity and radiosensetivity multiplier for the treatment of head and neck cancers.
